# Burden of Acute Respiratory Infections Among Under-Five Children in Relation to Household Wealth and Socioeconomic Status in Bangladesh

**DOI:** 10.3390/tropicalmed4010036

**Published:** 2019-02-12

**Authors:** Sanni Yaya, Ghose Bishwajit

**Affiliations:** Faculty of Social Sciences, School of International Development and Global Studies, University of Ottawa, Ottawa, ON K1N 6N5, Canada

**Keywords:** acute respiratory infections, fever, dyspnea, Bangladesh Demographic and Health Survey, socioeconomic status, household wealth

## Abstract

Acute respiratory infections (ARIs), as a group of diseases and symptoms, are a leading cause of morbidity and mortality among under-five children in tropical countries like Bangladesh. Currently, no clear evidence has been published on the prevalence and socioeconomic correlates of ARIs in Bangladesh. In this regard, we carried out this study with the aim of assessing the prevalence and the socioeconomic predictors of ARIs among children aged 0–59 months, with a special focus on socioeconomic status and wealth-related indicators. Cross-sectional data on 32,998 mother-child (singleton) pairs were collected from six rounds of Bangladesh Demographic and Health Surveys (BDHS 1997–2014). The outcome variable were presence of the common symptoms of ARIs, fever and dyspnea, during the previous two weeks, which were measured based on mothers’ reports about the symptoms of these conditions. Explanatory variables included maternal demographic and socioeconomic factors such as age, education, occupation, wealth quintile, and child’s age and sex. The prevalence and predictors of ARIs were measured using descriptive and multivariate regression methods. The prevalence of both fever (31.00% in 1997 vs. 36.76% in 2014) and dyspnea (39.27% in 1997 vs. 43.27% in 2014) has increased gradually since 1997, and tended to be higher in households in the lower wealth quintiles. Multivariable analysis revealed that higher maternal educational status, access to improved water and sanitation facilities, and living in households in higher wealth quintiles had protective effects against both fever and dyspnea. Findings suggested a significantly negative association between lacking access to improved water and sanitation and use of biomass fuel with ARI symptoms. However, no sex difference was observed in these associations. Based on the findings, childhood ARI prevention strategies should address the risk factors stemming from parental socioeconomic marginalisation, household water and sanitation poverty, and use of unclean fuel.

## 1. Introduction

With the sixth largest population on earth, Bangladesh has the eighth largest population in poverty [1], and the highest percentage of the population below the national poverty line among all South Asian countries [2]. Since its independence in 1971, the country has made remarkable strides in reducing extreme poverty and promoting key population health indicators such as maternal and child mortality, and providing access to basic public health services [3,4]. Bangladesh continues to face overwhelming challenges in meeting the basic healthcare needs of the population. The situation is particularly challenging among vulnerable groups of the population, such as women and children [5,6,7,8]. According to the United Nations International Children’s Emergency Fund (UNICEF), about half of all Bangladeshi children (~33 million) are living in poverty, with one-quarter being deprived of basic needs including food, education, health, and sanitation [9]. The impact of economic poverty on child health is further aggravated by chronic water and sanitation insecurity among the disadvantaged populations [10,11,12,13]. The World Health Organization (WHO) has estimated that 60% of the population in Bangladesh lacks access to improved sources of water and sanitation [14]. The persisting water and sanitation poverty represent a serious challenge for promoting child health in Bangladesh [10,15,16,17].

The entrenched economic, water, and sanitation poverty is reflected in the high burden of infectious diseases of poverty such as acute respiratory infections (ARIs), which represent the largest share of disease burden in Bangladesh [18]. ARIs, as a group of diseases, are classified as upper or lower respiratory tract infections whose negative effects can include infection, inflammation, and reduced lung function [19,20,21]. ARIs are the most common causes of morbidity and mortality among under-five children (except for neonates) [19]. Evidence from studies in India and sub-Saharan African countries suggests that poverty influences child health and disease outcomes, including ARIs, through various direct and indirect pathways [22,23,24,25]. For many poor households in Bangladesh, especially in rural areas, lack of electricity and clean fuel are common concerns that are circumvented by the use of biomass fuel such as coal, solid domestic waste, dried leaves and wood, and often kerosene [26,27,28]. Biomass fuel burning is known to be a major cause of indoor air pollution because of the organic material emissions, such as nitrogen oxides, carbon monoxide, and hazardous air pollutants (HAPs), which thereby contribute to the higher burden of ARIs [27,29,30]. However, no research evidence has been published on these associations among under-five children in Bangladesh. To address this research gap, in the present study, we analysed several nationally-representative datasets from the Bangladesh Demographic and Health Survey. The main objective was to measure the prevalence of ARIs among under-five children during the last two decades. We examined sociodemographic patterns in the prevalence of ARIs, with special focus on household wealth status and lack of improved water and sanitation facilities.

## 2. Methods

### 2.1. Data Sources

The study is based on cross-sectional data from six rounds of Bangladesh Demographic and Health Survey conducted between 1997 and 2014. The surveys are conducted by the National Institute of Population Research and Training of Bangladesh and Mitra and Associates with financial support provided by the U.S. Agency for International Development (USAID) and technical assistance by ICF (Inner City Fund) International of Calverton (NY), USA. The surveys are nationally representative and employ multistage sampling strategies for data collection. In general, the sampling frame consists of clusters (known as primary sampling units (PSUs) or enumeration areas) covering both urban and rural areas across the country. Afterward, the PSUs are stratified into homogenous subgroups (Strata) to ensure the representativeness of the sample. Households are then randomly selected from each stratum to interview eligible men and women. Further details of the sampling strategy, response rates, and questionnaires were published previously [31,32].

### 2.2. Measures

The outcome variable was the occurrence of ARI symptoms during the previous two weeks: (1) fever and (2) dyspnea for the youngest child in the household. This was assessed by asking the mothers about: (1) child having a fever during the last two weeks, and (2) child suffering from short, rapid breaths during last two weeks [22,25,30]. The answers were categorized as yes or no (no or do not know). In this paper, the term ARI refers to symptoms of ARIs.

Depending on the availability of the datasets, as well their theoretical relationship known from the literature [22,23,25,33], the following maternal demographic and socioeconomic (e.g., education and occupation) [34] and child-level variables were selected as the potential predictors of ARI: maternal age (15–19, 20–24, 25–29, or 30–34); residency (urban/ rural); region (Barisal Chittagong, Dhaka, Khulna, Rajshahi, Rangpur, or Sylhet); literacy level (no education, primary, secondary, higher); religion (Islam, other); sanitation (unimproved, improved); water (unimproved, improved); cooking fuel (unclean, clean); wealth quintile (poorest, poorer middle, richer, richest); employment (unemployed, employed); parity (2, 4, 5); age of child (0–11, 12–23, 24–35, 36–47, 48–59); and child’s sex (male/ female). Cooking fuel was categorized as clean if electricity, biogas, or liquefied petroleum gas were used, and unclean if kerosene or wood were used. Household wealth quintile was calculated based on scores of household possession of durable goods (e.g., television, refrigerator) using principal component analysis. The final scores were categorized into quintiles (poorest: q1, poorer: q2, middle: q3, richer: q4; and richest: Q5) so that higher quintiles represent better wealth status [35].

### 2.3. Data Analysis

Data were analysed with Stata Corp (College Station, Texas) version 14. Datasets were first cleaned, checked for missing values, outliers, multicollinearity issues (provided in the Appendix, Table A2), and merged in order to perform pooled analysis. Then, the dataset was prepared for cluster analysis by accounting for sampling design, sampling strata, and weight. From this point on, all the analyses were performed using the *survey* command. Sample characteristics are described by percentages with 95% confidence intervals (CIs). The prevalence of fever and dyspnea stratified by age groups and wealth quintile are presented as bar charts for all six rounds. As the outcome variables were dichotomous, binary logistic regression methods were applied to assess the relationship between fever and dyspnoea. Regression models for both of the outcome measures were stratified by child’s sex, and are presented as odds ratios and 95% CIs. All tests are two-tailed and were considered significant at alpha value of 5%.

For ethical clearance, all participants provided informed consent prior to participating in the survey. Further approval was not necessary for this study as the data were secondary and are available in public domain in anonymized form.

## 3. Results

### 3.1. Sample Characteristics

Basic sociodemographic characteristics of the sample population are summarised in Table A1 (available in the Appendix). The combined prevalence of fever among boys and girls was 51.6% (95% CI = 50.5–52.6) and 48.4% (95% CI = 47.4–49.5) respectively- and that of dyspnoea was 52.7% (95% CI = 51.0–54.3) and 47.3% (95% CI = 45.7–49.0)—respectively. The sex differences were not statistically significant. In 2014—the overall prevalence of fever was 36.76% (95% CI = 34.9–38.1) and that of and dyspnoea was 43.27% (95% CI = 42.4–44.6)—both being higher than their 1997 levels: fever 31.0% (95% CI = 29.9–32.4) and dyspnoea at 39.27% (95% CI = 38.2–41.1).

**Figure 1:**
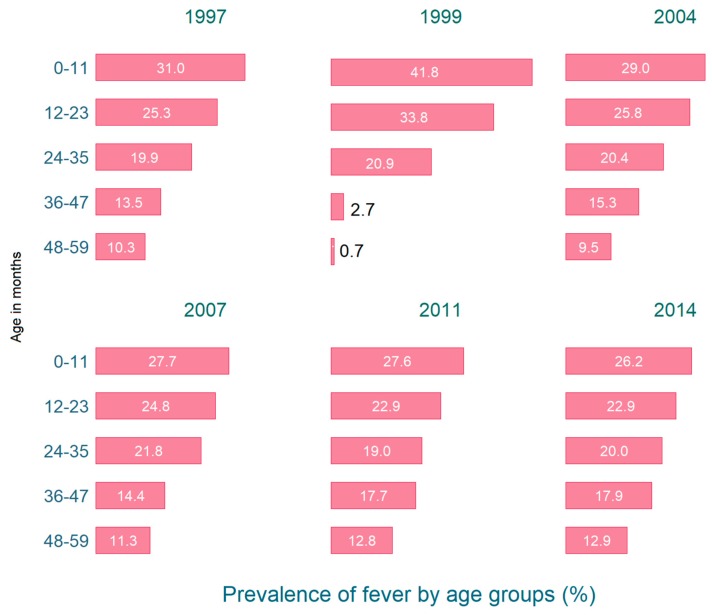
Prevalence of fever among under-five children in Bangladesh 1997–2014.

Figure 1 illustrates the prevalence of fever among under-five children for all the survey years. Since 1997, the prevalence declined, albeit slowly, among the younger age groups aged 0–11 and 12–23 months, but increased for the older children aged 36–47 and 48–59 months.

**Figure 2:**
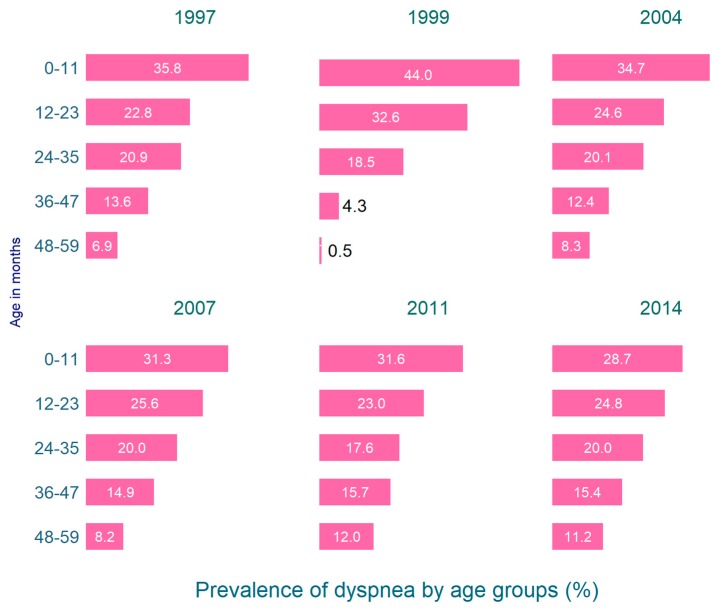
Prevalence of dyspnoea among under-five children in Bangladesh 1997–2014.

Figure 2 illustrates the prevalence of dyspnoea among under-five children for all the survey years. Since 1997, the prevalence declined only among the youngest groups aged 0–11 months and increased for the older children. During 1997–2014, the largest increase was observed in the highest age group of 48–59 months.

**Figure 3:**
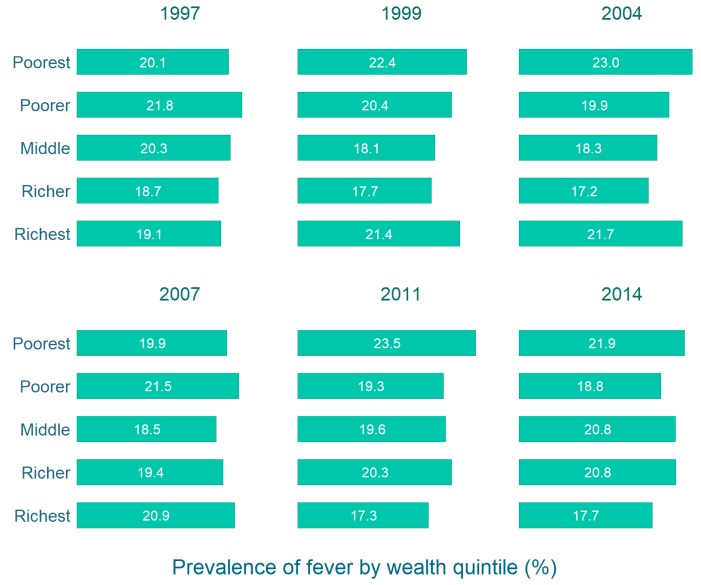
Prevalence of fever among under-five children by household wealth quintile in Bangladesh 1997–2014.

As shown in Figure 3, in 1997 the prevalence of fever was relatively higher in the lower wealth quintiles (poorer and poorest) than the higher quintiles (richer and richest). The pattern was somewhat similar for most of the surveys; the combined prevalence in the higher wealth quintiles was constantly lower than in the lower quintiles. This difference was statistically significant (*p* < 0.05).

**Figure 4:**
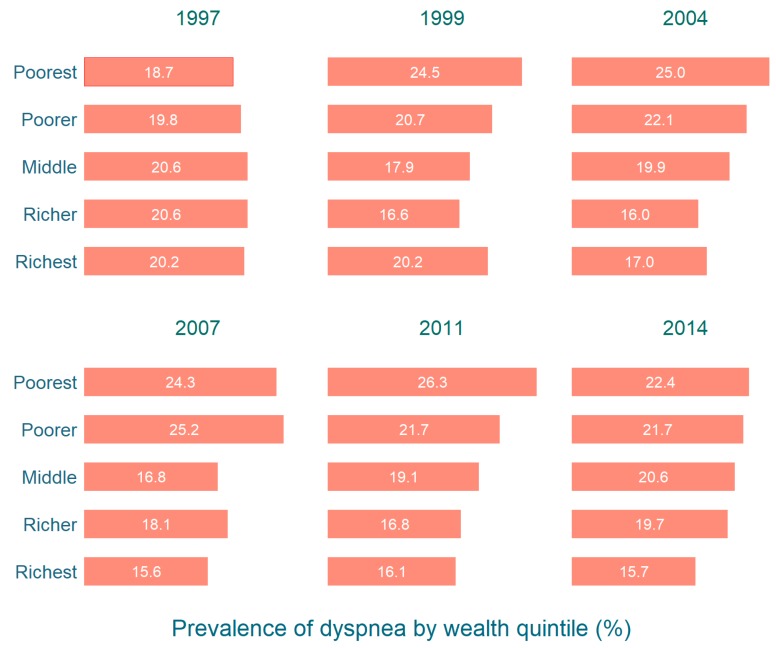
Prevalence of dyspnoea among under-five children by household wealth quintile in Bangladesh 1997–2014.

Similar to our findings on fever, Figure 4 shows that the prevalence of dyspnoea was generally higher in the lower wealth quintiles (poorer and poorest) than in the higher quintiles (richer and richest) for all survey years except for 1997. This difference was also statistically significant (*p* < 0.05).

### 3.2. Multivariable Analysis

The odds ratios (ORs) of the associations between the predictor variables with fever, dyspnea, and both fever and dyspnoea are presented in Table 1, Table 2 and Table 3, respectively. Mother’s age and religion did not show any significant association with fever. However, place of residency, region (for Khulna and Rajshahi only), education, and employment appeared to be significant predictors of fever and dyspnoea. The odds of fever were lower in Khulna (OR = 0.850, 95% CI = 0.723–0.999) but higher in the Rajshahi district (OR = 1.163, 95% CI = 1.001–1.352) (Table 1), whereas that of dyspnoea was lower in Rajshahi (OR = 0.770, 95% CI = 0.607,0.976) (Table 2). The odds of fever (OR = 0.809, 95% CI = 0.679–0.963) and dyspnoea (OR = 0.584, 95% CI = 0.384–0.890) were significantly lower when mothers had higher education levels. Children of unemployed mothers had higher odds of fever (OR = 1.117, 95% CI = 1.016–1.227), but not of dyspnoea. However, most of these associations lost their significance after stratifying by child’s sex. The odds of fever were lower for households in the higher wealth quintiles (e.g., OR = 0.824, 95% CI = 0.701–0.968 for the highest wealth quintile), and with access to improved water (OR = 0.856, 95% CI = 0.659–0.988) and sanitation (OR = 0.738, 95% CI = 0.652–0.932). Similar findings were found for dyspnoea and for the combined association of fever and dyspnea (Table 3). Using clean fuel (OR = 0.648, 95% CI = 0.464–0.903) and having access to improved sanitation (0.644 95% CI = 0.466–0.890) were associated lowers odds of having ARI symptoms. Compared with the poorest households, children in the richer (OR = 0.581, 95% CI = 0.352–0.959) and richest (0.592, 95% CI = 0.357–0.980) households had lower odds of having ARI symptoms.

## 4. Discussion

Acute respiratory infections (ARIs) are a leading cause of morbidity and mortality among under-five children in low-income countries like Bangladesh. Evidence suggests that in 1997–2001, ARIs including pneumonia were major contributors to hospitalization (40%) among under-five children at primary public care facilities in rural Bangladesh [35]. The findings of the present study are in line with the existing evidence that a remarkable proportion of the under-five children in Bangladesh continue to suffer from ARIs. As the findings indicate, the prevalence of fever and dyspnea has increased over the last 15 years, especially among children aged three to five years old. During 1997–2014, the overall prevalence has slowly but steadily risen, despite a net decline in the prevalence among infants (<12 months). Although previous studies reported significant sex differentials in childhood morbidity and mortality in Bangladesh [34,36], the present study suggests that the male-female disparity is not significant for ARIs.

A high ARI burden was reported in several sub-national studies among South Asian children including Bangladesh (72% in 2001, urban slums of Dhaka) [37] and India (59.1% in 2013–2014, Puducherry) [38]. ARI-attributed mortality rates were found to be high in Nepal (20–30%) [39] and Pakistan (20–30%) [40]; however, corresponding estimates are currently not available for Bangladesh. Existing studies suggest that the health impacts of the high disease burden among South Asian children are further exacerbated by sub-optimal care seeking behavior, especially among the poorest households [41,42]. In light of the past and current findings, ARIs remain a common health issue among under-five children in Bangladesh, which requires urgent attention from public health stakeholders.

Apart from the high prevalence, the findings also highlight the significant demographic and socioeconomic pattern in the occurrence of ARIs. The odds of fever or dyspnea did not differ noticeably across maternal age groups; however, being an older child appeared to be a protective factor against both fever and dyspnea. Maternal community level factors, such as rural residency and certain regional disparities, were observed in the odds of fever. For instance, compared to those based in Barishal district, the odds of fever were lower in Khulna but higher in Rajshahi, whereas the odds of dyspnea were lower in Rajshahi only. However, the association between region and ARIs was not consistent across the districts. Regarding maternal socioeconomic characteristics, having higher education and living in higher wealth quintile households were inversely associated with fever and dyspnea, indicating a protective effect of better maternal socioeconomic status (SES) on child’s experience of ARIs.

The beneficial effect of higher SES on child’s health outcomes is generally attributed to better living conditions, better nutritional status, and access to healthcare services. Parental material hardship can affect child health and exposure to illness through various pathways. For instance, financial stress is strongly correlated with child undernutrition, poor cognitive development, and weakened immune system, so can increase the vulnerability to infectious diseases. Children in financially well-off families are more likely to enjoy healthy and secure living facilities with greater access to health-promoting conditions in comparison to those from impoverished families [43]. Several other environmental aspects of poverty, such as inability to afford improved water, sanitation, and hygiene facilities (WASH) and clean cooking fuel, are also strong predictors of acute illnesses due to their link with important risk factors such as of fecal contamination of food, drinking water, utensils, and child’s toys [44].

This study reported the prevalence of ARIs among under-five children in Bangladesh, and their association with maternal and household socioeconomic indicators using nationally representative samples. Th information generated from this study should increase the awareness about this ever-growing health issue and help policy makers to implement appropriate policy measures. Our findings have the potential to inspire national WASH and poverty reduction policies in an effort to improve child poverty-reduction and health-promotion strategies. Bangladesh is also a highly disaster-prone country and is vulnerable to climate change, which are important risk factors for ARIs [45]. Growing evidence in this area of public health can help with the development of effective planning, coping, and intervention mechanisms. Apart from our important contributions, our study has several limitations. First, the symptoms of ARIs were reported by mothers and were not the result of objective measurement. The variables were self-reported, and are hence subject to reporting bias. Second, there were no data available on household hygiene practices, which is a key predictor of infectious diseases among populations of all ages. There were no data on whether the children were suffering from any specific illness; therefore, it is possible that the symptoms of fever and dyspnea resulted from conditions other than ARIs. However, this gap is expected to be overcome, to a great extent, by the inclusion of water and sanitation variables. Lastly, the data were secondary, and thus no causal inferences can be made about the associations.

## 5. Conclusions

Since 1997, there has been a slow but steady increase in the prevalence of ARI symptoms, such as fever and dyspnea, among under-five children in Bangladesh. Apart from the high prevalence, our study provides several important findings regarding the socioeconomic predictors of these conditions that might be of interest for child health- and WASH-related stakeholders in the country. Higher maternal educational status and employment status showed protective effects against suffering from fever. As expected, lack of improved WASH facilities was significantly associated higher odds of ARI. Of particular concern was the higher burden of ARIs in households in the lower wealth quintiles. Given these findings, we suggest that child-health-related sustainable development programs in Bangladesh should try to emphasize maternal socioeconomic status and ensuring better environmental conditions, such as optimum WASH coverage and use of clean fuel, especially among the most marginalised communities. More studies are required to investigate the broader macroeconomic and sociocultural factors that may underlie the constantly high prevalence of ARIs among under-five children.

## Figures and Tables

**Table 1:** Predictors of fever among under-five children in Bangladesh, Bangladesh Demographic and Health Survey 1997–2014.

	Overall	Male	Female
**Age (15–19)**	1	1	1
20–24	1.041	0.932	1.166
	(0.923, 1.175)	(0.786, 1.105)	(0.981, 1.386)
25–29	0.945	0.886	1.019
	(0.831, 1.075)	(0.738, 1.064)	(0.849, 1.223)
30+	0.987	0.910	1.076
	(0.862, 1.131)	(0.750, 1.104)	(0.889, 1.303)
**Residency (Urban)**	1	1	1
Rural	0.889 *	0.877	0.899
	(0.807, 0.980)	(0.765, 1.006)	(0.782, 1.032)
**Region (Barisal)**	1	1	1
Chittagong	1.133	1.458 ***	0.895
	(0.980, 1.310)	(1.186, 1.794)	(0.728, 1.100)
Dhaka	0.965	1.161	0.813
	(0.831, 1.120)	(0.939, 1.436)	(0.658, 1.004)
Khulna	0.850 *	0.958	0.765 *
	(0.723, 0.999)	(0.759, 1.208)	(0.610, 0.960)
Rajshahi	1.163 *	1.391 **	0.994
	(1.001, 1.352)	(1.123, 1.722)	(0.803, 1.230)
Rangpur	1.011	1.332 *	0.766 *
	(0.862, 1.185)	(1.063, 1.668)	(0.609, 0.963)
Sylhet	1.133	1.512 **	0.850
	(0.950, 1.352)	(1.179, 1.938)	(0.660, 1.095)
**Education (None)**	1	1	1
Primary	1.031	0.999	1.078
	(0.922, 1.153)	(0.855, 1.167)	(0.917, 1.269)
Secondary	1.013	0.907	1.144
	(0.902, 1.138)	(0.772, 1.066)	(0.967, 1.353)
Higher	0.809 *	0.767 *	0.853
	(0.679, 0.963)	(0.601, 0.979)	(0.664, 1.096)
**Religion (Islam)**	1	1	1
Others	0.894	0.773	0.960
	(0.689, 1.216)	(0.552, 1.121)	(0.781, 1.181)
**Sanitation (Unimproved)**	1	1	1
Improved	0.738 **	0.977	0.900
	(0.652, 0.932)	(0.854, 1.116)	(0.784, 1.032)
**Water (Unimproved)**	1	1	1
Improved	0.856 **	1.035	0.982
	(0.659, 0.988)	(0.975, 1.565)	(0.702, 1.639)
**Fuel (Unclean)**	1	1	1
Clean	0.830 **	0.817	0.938
	(0.593, 0.991)	(0.631, 1.150)	(0.749, 1.176)
**Employed (Yes)**	1	1	1
No	1.117 *	1.231 **	1.002
	(1.016, 1.227)	(1.078, 1.405)	(0.874, 1.148)
**Wealth (Poorest)**	1	1	1
Poorer	0.947	0.869	1.035
	(0.835, 1.073)	(0.731, 1.034)	(0.863, 1.241)
Middle	0.940	0.855	1.019
	(0.825, 1.069)	(0.713, 1.025)	(0.846, 1.227)
Richer	0.869 *	0.831	0.913
	(0.757, 0.998)	(0.685, 1.009)	(0.749, 1.114)
Richest	0.824 *	0.748 *	0.913
	(0.701, 0.968)	(0.593, 0.942)	(0.726, 1.147)
**Child’s Age (0–11 months)**	1	1	1
12–23	0.973	0.938	1.002
	(0.870, 1.088)	(0.801, 1.099)	(0.855, 1.173)
24–35	0.803 ***	0.769 **	0.828 *
	(0.715, 0.902)	(0.654, 0.905)	(0.701, 0.979)
36–47	0.772 ***	0.713 ***	0.838 *
	(0.682, 0.873)	(0.599, 0.848)	(0.702, 1.000)
48–59	0.631 ***	0.645 ***	0.607 ***
	(0.550, 0.725)	(0.533, 0.780)	(0.497, 0.743)
**Sex (Male)**			
Female	0.911	NA	NA
	(0.705, 1.143)		
Nagalekerke-R^2^	0.341	0.419	0.368

Note: All models are adjusted for year of survey. Figures represent odds ratios with 95% confidence intervals, reference categories in () brackets. * *p* < 0.05, ** *p* < 0.01, *** *p* < 0.001.

**Table 2:** Predictors of dyspnea among under-five children in Bangladesh. Bangladesh Demographic and Health Survey 1997–2014.

	Overall	Male	Female
**Age (15–19)**	1	1	1
20–24	0.802	0.828	0.774
	(0.666, 1.065)	(0.641, 1.068)	(0.587, 1.019)
25–29	0.888	0.679 **	0.934
	(0.643, 1.464)	(0.513, 0.898)	(0.694, 1.257)
30+	0.921	0.940	0.914
	(0.745, 1.139)	(0.698, 1.265)	(0.671, 1.243)
**Residency (Urban)**	1	1	1
Rural	0.900	1.020	0.777 *
	(0.771, 1.051)	(0.823, 1.263)	(0.619, 0.975)
**Region (Barisal)**	1	1	1
Chittagong	1.020	1.131	0.923
	(0.812, 1.281)	(0.822, 1.557)	(0.664, 1.282)
Dhaka	0.983	1.150	0.839
	(0.774, 1.247)	(0.818, 1.616)	(0.598, 1.176)
Khulna	0.850	0.941	0.757
	(0.658, 1.098)	(0.656, 1.351)	(0.525, 1.092)
Rajshahi	0.770 *	0.776	0.760
	(0.607, 0.976)	(0.554, 1.086)	(0.542, 1.067)
Rangpur	0.789	0.887	0.688
	(0.609, 1.022)	(0.618, 1.273)	(0.471, 1.003)
Sylhet	0.950	1.154	0.763
	(0.710, 1.272)	(0.775, 1.720)	(0.492, 1.183)
**Education (None)**	1	1	1
Primary	1.051	1.214	0.899
	(0.881, 1.254)	(0.952, 1.547)	(0.692, 1.167)
Secondary	0.823 *	0.916	0.724 *
	(0.683, 0.992)	(0.707, 1.187)	(0.551, 0.951)
Higher	0.584 *	0.961	0.758
	(0.384, 0.890)	(0.639, 1.445)	(0.567, 1.013)
**Religion (Islam)**	1	1	1
Others	0.873	0.758	0.993
	(0.690, 1.105)	(0.547, 1.052)	(0.704, 1.401)
**Sanitation (Unimproved)**	1	1	1
Improved	0.724 **	1.009	0.836
	(0.595, 8.073)	(0.817, 1.244)	(0.672, 1.041)
**Water (Unimproved)**	1	1	1
Improved	0.820 *	0.941	1.014
	(0.672, 0.949)	(0.714, 1.518)	(0.667, 1.544)
**Fuel (Unclean)**	1	1	1
Clean	0.682 **	0.635 *	0.717
	(0.520, 0.894)	(0.433, 0.930)	(0.485, 1.060)
**Employed (Yes)**	1	1	1
No	0.987	0.953	1.021
	(0.848, 1.149)	(0.772, 1.177)	(0.818, 1.275)
**Wealth (Poorest)**	1	1	1
Poorer	1.162	1.067	1.279
	(0.953, 1.416)	(0.813, 1.400)	(0.954, 1.715)
Middle	1.030	0.882	1.210
	(0.838, 1.266)	(0.663, 1.173)	(0.892, 1.640)
Richer	0.849 *	0.794	0.915
	(0.681, 0.960)	(0.583, 1.081)	(0.662, 1.265)
Richest	0.784 *	0.740	0.842
	(0.605, 0.916)	(0.514, 1.067)	(0.579, 1.224)
**Child’s Age (0–11 months)**	1	1	1
12–23	0.873	0.760 *	1.019
	(0.734, 1.038)	(0.596, 0.968)	(0.793, 1.308)
24–35	0.812 *	0.704 **	0.951
	(0.676, 0.976)	(0.547, 0.907)	(0.725, 1.248)
36–47	0.661 ***	0.521 ***	0.863
	(0.540, 0.809)	(0.394, 0.690)	(0.641, 1.160)
48–59	0.639 ***	0.600 **	0.693 *
	(0.508, 0.804)	(0.439, 0.821)	(0.492, 0.978)
**Sex (Male)**			
Female	0.946	NA	NA
	(0.835, 1.073)		
Nagalekerke-R^2^	0.613	0.441	0.468

Note: All models are adjusted for year of survey. Figures represent odds ratios with 95% confidence intervals; reference categories in () brackets. * *p* < 0.05, ** *p* < 0.01, *** *p* < 0.001. Goodness of fit of the regression models was assessed by Nagalekerke-R^2^ values that indicated moderate to good predictive capacity for all the models.

**Table 3:** Predictors of fever and dyspnea among under-five children in Bangladesh. Bangladesh Demographic and Health Survey 1997–2014.

	Overall	Male	Female
**Age (15–19)**	1	1	1
20–24	1.035	0.997	1.067
	(0.802, 1.335)	(0.695, 1.431)	(0.740, 1.537)
25–29	1.133	0.879	1.560 *
	(0.857, 1.498)	(0.597, 1.294)	(1.032, 2.358)
30+	1.321	1.037	1.711 *
	(0.979, 1.782)	(0.680, 1.581)	(1.107, 2.644)
**Residency (Urban)**	1	1	1
Rural	0.955	0.972	0.911
	(0.772, 1.181)	(0.723, 1.307)	(0.666, 1.246)
**Region (Barisal)**	1	1	1
Chittagong	1.033	1.640 *	0.592 *
	(0.745, 1.430)	(1.058, 2.541)	(0.357, 0.982)
Dhaka	1.083	1.719 *	0.664
	(0.770, 1.524)	(1.073, 2.754)	(0.394, 1.118)
Khulna	0.615 **	0.774	0.458 **
	(0.438, 0.862)	(0.493, 1.214)	(0.270, 0.778)
Rajshahi	0.915	1.122	0.702
	(0.654, 1.280)	(0.720, 1.749)	(0.414, 1.191)
Rangpur	0.819	1.261	0.489 *
	(0.574, 1.170)	(0.774, 2.054)	(0.284, 0.844)
Sylhet	2.185 **	3.306 ***	1.358
	(1.312, 3.638)	(1.670, 6.546)	(0.621, 2.970)
**Education (None)**	1	1	1
Primary	1.058	0.923	1.270
	(0.822, 1.363)	(0.649, 1.312)	(0.873, 1.847)
Secondary	1.138	1.028	1.337
	(0.876, 1.480)	(0.711, 1.484)	(0.911, 1.962)
Higher	0.892	1.282	0.653
	(0.619, 1.285)	(0.741, 2.220)	(0.392, 1.086)
**Religion (Islam)**	1	1	1
Others	0.792	0.699	0.844
	(0.588, 1.067)	(0.466, 1.050)	(0.540, 1.320)
**Sanitation (Unimproved)**	1	1	1
Improved	0.644 **	0.962	0.807
	(0.466, 0.890)	(0.712, 1.299)	(0.650, 1.003)
**Water (Unimproved)**	1	1	1
Improved	1.051	1.120	0.998
	(0.699, 1.580)	(0.648, 1.935)	(0.534, 1.863)
**Fuel (Unclean)**	1	1	1
Clean	0.648 *	0.565 *	0.716
	(0.464, 0.903)	(0.354, 0.901)	(0.439, 1.167)
**Employed (Yes)**	1	1	1
No	1.113	1.153	1.050
	(0.899, 1.379)	(0.855, 1.556)	(0.768, 1.435)
**Wealth (Poorest)**	1	1	1
Poorer	0.983	0.937	0.990
	(0.736, 1.312)	(0.619, 1.418)	(0.656, 1.494)
Middle	1.130	0.738	1.127
	(0.832, 1.537)	(0.487, 1.119)	(0.738, 2.933)
Richer	0.581 *	0.695	0.999
	(0.352, 0.959)	(0.446, 1.081)	(0.643, 1.553)
Richest	0.592 *	0.850	1.169
	(0.357, 0.980)	(0.624, 1.157)	(0.703, 1.946)
**Child’s Age (0–11 months)**	1	1	1
12–23	1.051	0.970	1.111
	(0.821, 1.345)	(0.685, 1.374)	(0.780, 1.583)
24–35	0.861	0.950	0.739
	(0.669, 1.107)	(0.664, 1.360)	(0.515, 1.062)
36–47	0.900	0.696	1.228
	(0.682, 1.189)	(0.481, 1.008)	(0.793, 1.902)
48–59	0.749	0.857	0.629 *
	(0.552, 1.016)	(0.557, 1.317)	(0.404, 0.980)
**Sex (Male)**	1		
Female	0.976	NA	NA
	(0.821, 1.161)		
Nagalekerke-R^2^	0.374	0.280	0.412

Note: All models are adjusted for year of survey. Figures represent odds ratios with 95% confidence intervals; reference categories in () brackets. * *p* < 0.05, ** *p* < 0.01, *** *p* < 0.001.

## Abbreviations

ARIs	Acute Respiratory Infections
BDHS	Bangladesh Demographic and Health Survey
WASH	Water, sanitation and hygiene facilities

## Appendix A

**Table A1:** Sample characteristics BDHS 1997–2014.

		**Fever (*N* = 30,731)**		***p*-value**	**Dyspnoea (*N* = 11,354)**		***p*-value**
		No	Yes		No	Yes	
**Maternal Indicators**							
**Age**							
15–19	15.2	15.5 (14.9–16.2)	16.6 (15.9–17.4)	<0.001	16.9 (15.9–18.0)	18.8 (17.6–20.1)	<0.001
20–24	32.5	32.0 (31.1–32.8)	33.5 (32.5–34.6)		33.8 (32.5–35.2)	33.3 (31.7–34.8)	
25–29	27.0	27.2 (26.5–27.9)	25.9 (24.9–26.8)		26.0 (24.8–27.3)	24.9 (23.4–26.4)	
30–34	25.3	25.3 (24.6–26.1)	24.0 (23.1–24.9)		23.2 (22.0–24.4)	23.0 (21.8–24.4)	
**Residency**							
Urban	29.2	21.2 (19.9–22.5)	19.7 (18.3–21.2)	<0.001	21.1 (19.5–22.8)	19.1 (17.5–20.8)	<0.001
Rural	70.8	78.8 (77.5–80.1)	80.3 (78.8–81.7)		78.9 (77.2–80.5)	80.9 (79.2–82.5)	
**Region**							
Barisal	11.4	6.1 (5.3–6.9)	6.3 (5.5–7.2)	<0.001	6.1 (5.2–7.1)	6.6 (5.5–7.8)	<0.001
Chittagong	17.9	19.8 (18.2–21.5)	23.3 (21.4–25.4)		22.7 (20.6–24.9)	24.7 (22.4–27.1)	
Dhaka	20.6	33.4 (31.3–35.5)	30.0 (27.8–32.4)		30.8 (28.5–33.1)	28.5 (26.1–31.1)	
Khulna	12.3	11.1 (10.0–12.3)	8.6 (7.6–9.6)		9.7 (8.6–11.0)	9.6 (8.3–10.9)	
Rajshahi	18.7	18.2 (16.6–19.9)	19.5 (17.7–21.4)		19.7 (17.7–21.8)	18.4 (16.4–20.6)	
Rangpur	13.2	8.5 (7.6–9.5)	8.6 (7.6–9.7)		8.1 (6.9–9.6)	8.7 (7.5–10.0)	
Sylhet	6.0	3.1 (2.6–3.6)	3.7 (3.0–4.6)		2.9 (2.3–3.8)	3.6 (2.8–4.6)	
**Literacy Level**							
No Education	29.1	30.6 (29.5–31.8)	30.3 (29.1–31.6)	<0.001	30.0 (28.5–31.5)	31.9 (30.2–33.6)	<0.001
Primary	29.2	28.5 (27.6–29.3)	29.9 (28.8–31.0)		28.4 (27.1–29.7)	32.3 (30.6–33.9)	
Secondary	34.0	33.6 (32.6–34.7)	34.4 (33.1–35.7)		35.1 (33.5–36.6)	31.3 (29.7–33.0)	
Higher	7.7	7.3 (6.8–7.8)	5.4 (4.9–5.9)		6.6 (5.9–7.3)	4.6 (3.9–5.3)	
**Religion**							
Islam	90.0	90.4 (89.4–91.3)	92.0 (91.0–93.0)	0.021	91.4 (90.2–92.4)	91.0 (89.6–92.2)	0.014
Other	10.0	9.6 (8.7–10.6)	8.0 (7.0–9.0)		8.6 (7.6–9.8)	9.0 (7.8–10.4)	
**Sanitation**							
Unimproved	45.6	47.9 (46.6–49.2)	49.3 (47.7–50.8)		47.8 (46.0–49.6)	51.6 (49.6–53.6)	
Improved	54.4	52.1 (50.8–53.4)	50.7 (49.2–52.3)		52.2 (50.4–54.0)	48.4 (46.4–50.4)	
**Water**							
Unimproved	6.8	7.0 (6.5–7.6)	6.4 (5.7–7.1)	<0.001	6.3 (5.5–7.1)	6.6 (5.5–7.3)	<0.001
Improved	93.2	93.0 (92.4–93.5)	93.6 (92.9–94.3)		93.7 (92.9–94.5)	93.4 (92.2–95.8)	
**Cooking Fuel**							
Unclean	89.0	88.0 (86.6–89.2)	90.9 (89.6–92.0)		88.8 (87.1–90.3)	92.5 (91.1–93.6)	
Clean	11.0	12.0 (10.8–13.4)	9.1 (8.0–10.4)		11.2 (9.7–12.9)	7.5 (6.4–8.9)	
**Wealth Quintile**							
Poorest	20.0	20.3 (19.3–21.4)	23.1 (21.9–24.4)	<0.001	20.9 (19.6–22.3)	25.7 (24.0–27.5)	<0.001
Poorer	20.0	20.5 (19.7–21.3)	20.6 (19.6–21.7)		19.6 (18.4–20.9)	22.1 (20.6–23.6)	
Middle	18.8	18.5 (17.8–19.3)	19.8 (18.6–21.0)		19.1 (17.8–20.5)	19.3 (18.0–20.7)	
Richer	19.4	19.6 (18.8–20.5)	19.0 (18.0–20.0)		19.7 (18.5–21.0)	17.5 (16.2–18.9)	
Richest	21.8	21.1 (19.9–22.2)	17.5 (16.3–18.7)		20.7 (19.2–22.3)	15.4 (14.2–16.8)	
**Employment**							
Unemployed	75.8	75.3 (74.0–76.6)	75.1 (73.6–76.6)	0.021	74.8 (73.0–76.6)	76.9 (74.9–78.8)	0.017
Employed	24.2	24.7 (23.4–26.0)	24.9 (23.4–26.4)		25.2 (23.4–27.0)	23.1 (21.2–25.1)	
**Parity**							
2	59.0	59.4 (58.4–60.4)	58.1 (57.0–59.3)	0.041	59.4 (57.9–60.9)	57.9 (56.3–59.5)	0.018
4	27.6	27.1 (26.4–27.9)	28.5 (27.5–29.4)		28.4 (27.2–29.7)	27.6 (26.3–29.0)	
5	13.3	13.5 (12.8–14.2)	13.4 (12.6–14.3)		12.2 (11.2–13.2)	14.5 (13.3–15.8)	
**Age of Child**							
0–11	24.3	23.0 (22.3–23.7)	27.3 (26.3–28.4)	<0.001	25.6 (24.4–26.9)	32.0 (30.4–33.7)	0.024
12–23	21.0	20.3 (19.5–21.1)	23.4 (22.4–24.4)		22.4 (21.1–23.7)	23.5 (22.0–25.0)	
24–35	20.9	20.5 (19.8–21.2)	19.7 (18.9–20.6)		20.3 (19.1–21.5)	18.7 (17.5–20.0)	
36–47	18.9	19.8 (19.1–20.5)	16.5 (15.6–17.4)		17.7 (16.6–18.9)	14.4 (13.2–15.6)	
48–59	14.9	16.5 (15.8–17.2)	13.0 (12.0–14.0)		14.0 (12.7–15.4)	11.4 (10.4–12.6)	
**Child’s Sex**							
Male	50.9	51.1 (50.3–52.0)	51.6 (50.5–52.6)	0.128	51.3 (49.9–52.7)	52.7 (51.0–54.3)	0.191
Female	49.1	48.9 (48.0–49.7)	48.4 (47.4–49.5)		48.7 (47.3–50.1)	47.3 (45.7–49.0)	

Note: confidence intervals shown in parentheses.

**Table A2:** Multicollinearity tests.

**Multicollinearity Test for Outcome 1 (Fever):**	
Variable	VIF
Water	8.91
Parity	8.23
Age	7.66
Residency	7.44
Wealth	6.96
Child’s Sex	5.69
Region	4.77
Education	4.43
Sanitation	3.80
Fuel	1.55
Occupation	1.34
Religion	1.13
**Multicollinearity test for outcome 2 (dyspnoea):**	
Variable	VIF
Water	8.46
Parity	7.16
Age	5.31
Residency	5.14
Wealth	4.96
Child’s Sex	4.12
Region	3.17
Education	3.23
Sanitation	2.17
Fuel	1.55
Occupation	1.32
Religion	1.13
